# Circular RNAs and esophageal cancer

**DOI:** 10.1186/s12935-020-01451-0

**Published:** 2020-08-03

**Authors:** Xiaoqing Zhang, Ning Lu, Li Wang, Yixuan Wang, Minna Li, Ying Zhou, Honglin Yan, Manli Cui, Mingxin Zhang, Lingmin Zhang

**Affiliations:** 1 Department of Gastroenterology, The First Affiliated Hospital of Xi’an Medical University, No. 48 Feng Hao West Road, Xi’an, 710077 Shaanxi China; 2 Department of Scientific Research, The First Affiliated Hospital of Xi’an Medical University, Xi’an, Shaanxi China; 3 Shaanxi University of Traditional Chinese Medicine, Xianyang, 712046 Shaanxi China; 4 Department of Anesthesiology, First Affiliated Hospital, Xi’an Jiaotong University, No. 277 Yanta West Road, Xi’an, 710061 Shaanxi China

**Keywords:** Circular RNA, Esophageal cancer, Sponge effect, Biomarker

## Abstract

As a new kind of RNA, circular RNA (circRNA) is a endogenous non-coding RNA with circular structure, which has the characteristics of universality, stability, conservatism and specificity. CircRNA can specifically bind to microRNAs (miRNAs) in the form of competitive endogenous RNA, thus directly or indirectly regulating the expression of related genes. In addition to the role of sponge, circRNA also regulates parental gene expression, transcriptional translation and protein modification; and it can be used as a biomarker to develop potential diagnosis and treatment methods and evaluate prognosis. Due to changes in dietary habits and genetic factors, the morbidity and mortality of esophageal cancer (EC) in the world are still high, and are prone to early metastasis. Although the diagnosis and treatment techniques have been improved in recent years, the early diagnosis of EC is not common, and the 5-year survival rate of patients is still very low. This article reviews the function and significance of circRNA and discusses the research progress of circRNA as biomarkers in EC.

## Background

Esophageal cancer (EC) is one of the most common malignant tumors in the world, with high morbidity and mortality in China. The histological types of esophageal cancer mainly include esophageal squamous cell carcinoma (ESCC) and esophageal adenocarcinoma (EAC), 90% of which are ESCC [1]. The incidence rate of EC in China accounts for more than 70% of the world, with the characteristics of unobvious geographical distribution and high-risk areas, especially in Henan Province [2]. With the rapid development of radiotherapy, chemotherapy and surgery in recent years, great progress has been made in the treatment of EC, but early diagnosis is not common and is still an urgent problem to be solved. In addition, like other cancers, EC is characterized by epigenetic abnormalities and disorders in signaling pathways, but the molecular and genetic mechanisms of EC progression are not fully understood [3]. The prognosis of EC is still very poor, with a 5-year overall survival rate of 20% to 30% [4]. Therefore, early identification of biomarkers for prediction and prognosis is essential for improving current treatment strategies and prolonging 5-year survival rates.

Non-coding RNA (ncRNA) is a kind of RNA which is transcribed from the genome, does not encode protein, and can perform various biological functions at the RNA level. There are many kinds of ncRNA. At present, there are mainly long-chain non-coding RNA (lncRNA), micro-RNA (miRNA), circular RNA (circRNA) and so on. LncRNA is a kind of RNA whose transcript length is more than 200 bp. MiRNA is a kind of small RNA with a length of about 18 to 25 nucleotides. LncRNA and miRNA both belong to linear ncRNAs. In previous studies, ncRNA studies are more focused on linear ncRNAs, which shown that these linear ncRNAs have a variety of functions in physiological and pathological processes. In recent years, circRNA has attracted much attention as a potential biomarker for the treatment of different cancers [5, 6]. However, there are few studies on the regulation and clinical application of circRNA in EC. As a unique circular RNA, it was initially considered as an accidental by-product or “transcriptional noise” caused by low abundance and low functional potential resulting from errors in post-transcriptional processing [7]. With the rapid development of high-throughput sequencing, especially RNA sequencing, it is found that circRNA is not a sequencing product, but generally expressed in human genes [8], and can be verified by quantitative PCR [9]. CircRNA is a special kind of endogenous non-coding RNA, which forms a closed ring structure by covalent bond connection between 3′ and 5′ ends by trans-splicing. This closed ring structure is insensitive to nucleic acid exonuclease and highly stable [10]. More and more studies have shown that circRNA plays an important role in the progression of EC. This article reviews the function of circRNA and its role as biomarkers for the diagnosis, treatment and prognosis of EC.

## Functions of circRNAs

**Fig. 1:**
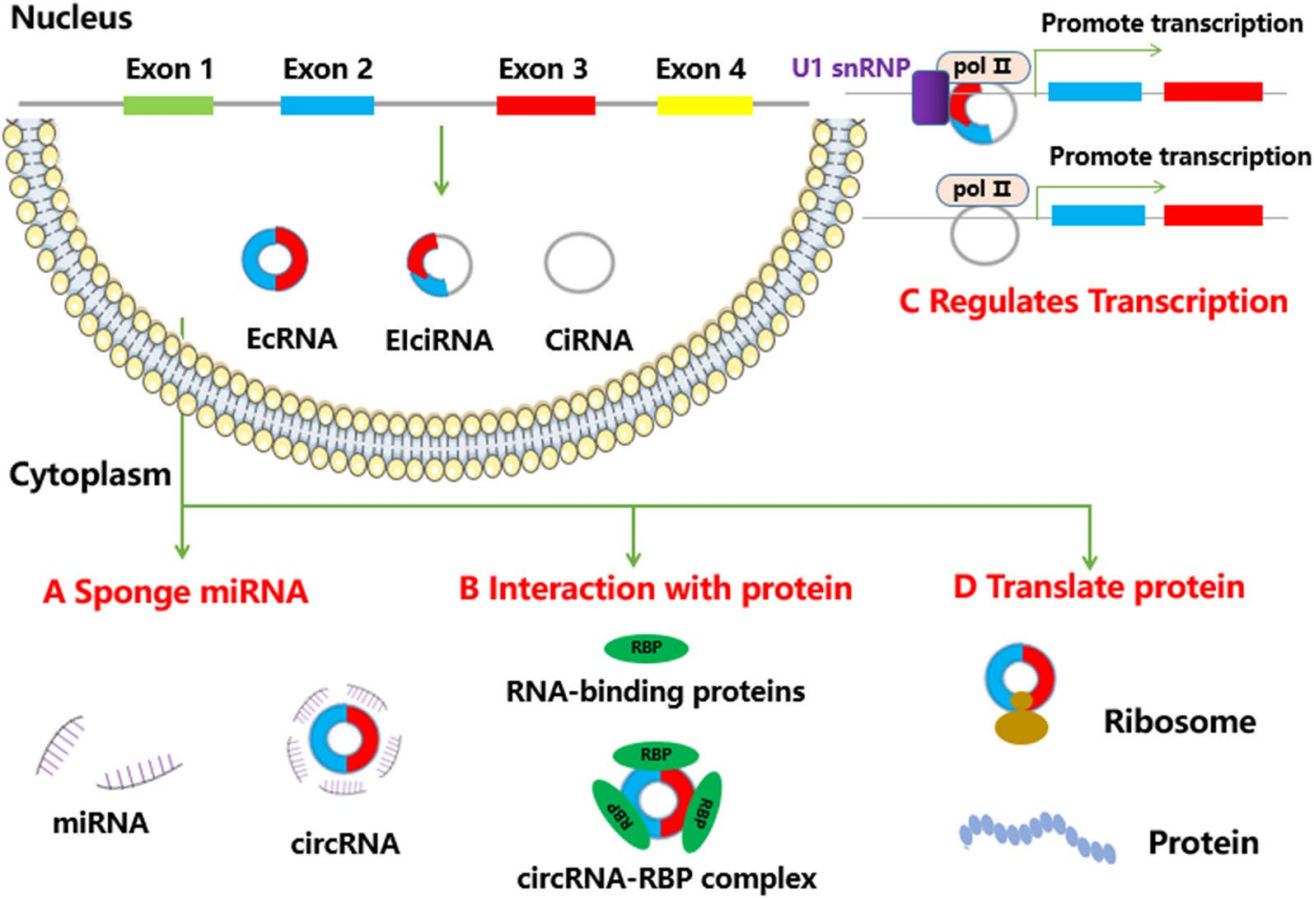
The functions of circRNAs. Four modes of circRNA functions are shown in the picture. **a** CircRNAs can act as miRNA sponges by competing for the binding of miRNA sequences, and mitigating the impact of miRNA-mediated regulation on gene expression. **b** CircRNAs can interact with proteins, by binding to other RNA-binding proteins (RBPs). **c** CircRNAs (EIciRNAs and CiRNAs) can enhance the expression of its parent genes through the formation of transcription complexes. **d** CircRNAs can be translated to create functional proteins

As a new type of RNA molecule, circRNA is a single-stranded ring molecule produced by the linear precursor mRNA (pre-mRNA) in a non-classical splicing square, which is transcribed by RNA polymerase II [11]. Its transcriptional efficiency is the same as that of linear RNA [10]. According to their different composition and cycling mechanism, the circRNA found so far can be simply divided into three categories: exon circRNA, intron circRNA and exon–intron circRNA. Because of its special structure, circRNA has the characteristics of universality, stability, conservatism and specificity [12]. Thus, circRNA becomes an ideal biomarker for the diagnosis, treatment and prognosis of EC. On the basis of sufficient research on the biogenesis of circRNA, more and more researches focus on its regulation. CircRNA can act as a miRNA “sponge”. MiRNAs can regulate gene expression by directly pairing with the target site in mRNAs, and is considered to be involved in a variety of biological and pathological processes, including cancer [13, 14]. Most circRNAs are mainly located in the cytoplasm [15], indicating that circRNAs may compete for endogenous RNAs and regulate miRNA activity by competing for miRNA binding sites [16, 17]. In addition to the “sponge” effect, circRNA also regulates parental gene expression, transcriptional translation and protein modification (Fig. 1) [18, 19]. At present, studies on the regulatory role of circRNAs in EC are still in its infancy. Therefore, further studies on circRNA not only help to clarify the molecular mechanism of esophageal carcinogenesis, but also provide the possibility for new molecules to be used as non-invasive diagnostic biomarkers.

### CircRNAs regulate gene expression as MiRNA sponge

One of the confirmed functions of circRNA is to regulate gene expression by acting as a miRNA “sponge” [17]. Compared with other competitive endogenous RNA (ceRNA), circRNA has a stronger ability to bind miRNAs and is called “super sponge”. Jiang et al. [20] constructed the ceRNA network in ESCC, Which linked 32 differentially expressed circRNAs and 98 differentially expressed mRNAs by 64 miRNAs. And helps to better understand circRNA-related mechanisms in ESCC. At present, the two classic examples of circRNA acting as a miRNA “sponge” are ciRS-7 (also known as CDR1as) and circ-SRY. It has been found that ciRS-7 is about 1.5 kb in length and contains more than 70 miR-7 binding sites [21]. Most of the binding sites bind to RNA-induced silencing complex (RISC), and RISC is formed by Ago2 protein and miR-7 [22]. In addition, it was found that the phenotype caused by miR-7 overexpression was similar to that caused by ciRS-7 gene knockout, suggesting that ciRS-7 may play an important role in pathophysiology through the miR-7/ciRS-7 axis [17, 23]. Similarly, circ-SRY is a circRNA originating from the sex-determining region Y (SRY). It has similar function to ciRS-7 [24] and contains 16 binding sites to miR-138, regulating the expression of miR-138 target genes [25]. miR-138 is associated with squamous cell carcinoma of the tongue and undifferentiated thyroid carcinoma, so the SRY gene may also be associated with human diseases [26].

### Interaction between circRNA and protein

In addition to the effect of circRNA on miRNA, another function of circRNA is its interaction with proteins. Previous studies have shown that circRNAs can regulate parental gene expression by affecting pre-mRNA. Ashwal-Fluss et al. found that there are conservative muscle-blind binding sites in circRNA (circMBL) and its flanking introns, which are firmly and specifically bound by MBL [11]. The regulation of MBL level strongly affects the biosynthesis of circMBL, which depends on MBL binding sites. Further data show that circRNAs can compete with pre-mRNA splicing for transcriptional function. As a regulator, exon circRNA plays the same role in the process of protein binding. It is reported that circ-Foxo3 is highly expressed in non-tumor cells and related to the cell cycle. The ectopic expression of circ-Foxo3 prevents the progression of cell cycle in G1 phase by forming circ-Foxo3-p21-CDK2 ternary complex [27]. Further, using molecular and cellular biological methods, it is concluded that ID-1, E2F1, FAK and HIF1α interact with circ-Foxo3 and remain in the cytoplasm, but no longer play their anti-aging and anti-stress effects, resulting in increased cell senescence [28]. In addition, an analysis shows that high levels of circPABPN1 block the binding of HuR to PABPN1 mRNA and inhibit the translation of PABPN1 [29].

### CircRNA regulates gene transcription

The regulation of circRNA at the transcriptional level may be the general function of the intron sequence circRNAs. Studies have shown that circRNA is abundant in the nucleus, in which ci-ankrd52 is mainly enriched in the transcriptional site of its parent genes, and is related to the extension mechanism of RNA pol II. circRNA acts as a positive regulator of RNA pol II transcription, indicating the cis-regulation of non-coding introns on the transcription of their parent genes [30]. It is reported that CiRNAs (intron circRNAs) and EIciRNAs (exonic-intronic circRNAs) could regulate protein production by transcriptional or post-transcriptional regulation of gene expression in the nucleus [31]. Another finding reveals a new role of circRNA in regulating nuclear gene expression, in which EIciRNA enhances the expression of its parent genes in cis and emphasizes the promotion of parental gene transcription through the formation of EIciRNAs-U1-snRNP complexes [32].

### CircRNA can be translated into protein

CircRNAs, similar to linear mRNAs, it can be used as a template for protein synthesis. It has been reported that eukaryotic ribosomes can initiate translation on circRNA, but only if the RNA contains an internal ribosome entry site element (IRES) [33]. A functional study shows that the inhibitory effect of FBXW7 protein on the malignant phenotype of human glioblastoma, proving that endogenous circRNA has translation ability and opens up a new field of circRNA regulation function [34].

## CircRNAs as biomarkers in EC

The expression pattern and characteristics of circRNA (universality, stability, conservatism and specificity) make it an ideal biomarker. Patients with EC lack effective diagnostic markers and therapeutic targets, which is part of the reason for their poor prognosis. Therefore, there is an urgent need to find biomarkers or therapeutic targets to improve the clinical prognosis of EC. At present, it has been observed that some circRNAs can be used as diagnostic and prognostic markers in patients with EC. Table 1 summarizes several studies that show the role of circRNAs as biomarkers in EC.

**Table 1:** The role of circRNAs as biomarkers

CircRNAs	Diagnosis	Prognosis	Radioresistance	References
circ_0004771	+	+		[35]
circGSK3β	+	+		[37]
circ_0043898	+	+	+	[51]
circ_100367		+	+	[52]
circ_001059		+	+	[53]
circ_000167		+	+	[53]
circ_0000654		+		[38]
circ-Foxo3		+		[50]
circPVT1		+		[39]
circ_0006168		+		[40]
circ_0004370		+		[41]
circ_ 0001946		+		[64]
circ-SMAD7		+		[49]
circ-TTC17		+		[42]
ciRS-7		+		[54, 65]
circ_0067934		+		[43]
circ_0006948		+		[44]
circ_0030018		+		[45]
circ _100876		+		[46]
circ-DLG1		+		[47]
circ-SLC7A5		+		[48]
circ UBAP2		+		[55]
circ RAD23B		+		[60]
cZNF292		+		[61]
circ FNDC3B		+		[56]
circ PRKCI		+		[59]
circ_0000337		+		[66]
circ_100873		+		[57]
circ LARP4		+		[62]
circ ITCH		+		[58]

Accumulated evidence shows that circRNA is a potential biomarker of EC. The expression of circ_0004771 was up-regulated in plasma and tissues of patients with EC. QRT-PCR verification and ROC curve analysis showed that circ_0004771 had higher significance and better diagnostic value, and its expression level was significantly correlated with T grade (invasion of the primary tumor range) and vascular invasion, suggesting that circ_0004771 can be used as an index to judge the prognosis [35]. At present, carcinoembryonic antigen (CEA) is one of the most commonly used diagnostic markers for EC [36]. Studies have shown that the combined application of circGSK3β and CEA may provide a new and promising biomarker for the early diagnosis of ESCC. In order to further evaluate the predictive value of plasma circGSK3β level for postoperative clinical improvement and whether it can predict the recurrence and metastasis of ESCC, further research were carried out and the results showed that the level of circGSK3β in patients with recurrence/metastasis was significantly higher than that in patients without recurrence/metastasis 10 months after operation. It is suggested that the level of plasma circGSK3β may be a valuable predictor of recurrence/metastasis of EC [37]. Following results showed that the expressions of circ_0000654 [38], circPVT1 [39], circ_0006168 [40], circ_0004370 [41], circ-TTC17 [42], circ_0067934 [43], circ_0006948 [44], circ_0030018 [45], circ_100876 [46], circ-DLG1 [47] and circ-SLC7A5 [48] were up-regulated in EC and related to the poor prognosis of EC. However, the other group found that the expression of circ-SMAD7 in EC was significantly down-regulated and negatively correlated with tumor stage and lymph node metastasis [49], and the low expression of circ-Foxo3 was related to poor prognosis [50]. Through the evaluation of circRNA, it was found that circ_0043898 [51], circ_100367 [52], circ_001059 [53] and circ_000167 [53] were related to the radiosensitivity of EC cells, which we will discuss in the following part.

## Role and significance of CircRNAs in EC

In order to understand the role of circRNAs in the progress of EC, there have been a large number of research reports on circRNAs in EC. More and more studies have shown that in EC, circRNAs can be used as an oncogene or tumor suppressor to regulate the proliferation, migration, invasion, apoptosis, cell cycle, epithelial–mesenchymal transition (EMT) and radioresistance of EC.

### Regulation of circRNA on proliferation, migration, invasion and apoptosis of EC

The ability to regulate cell proliferation and apoptosis is very important in tumor therapy. Many literatures have shown that circRNAs mediates EC cell proliferation and apoptosis, or interaction, and regulates cell migration and invasion.

**Fig. 2:**
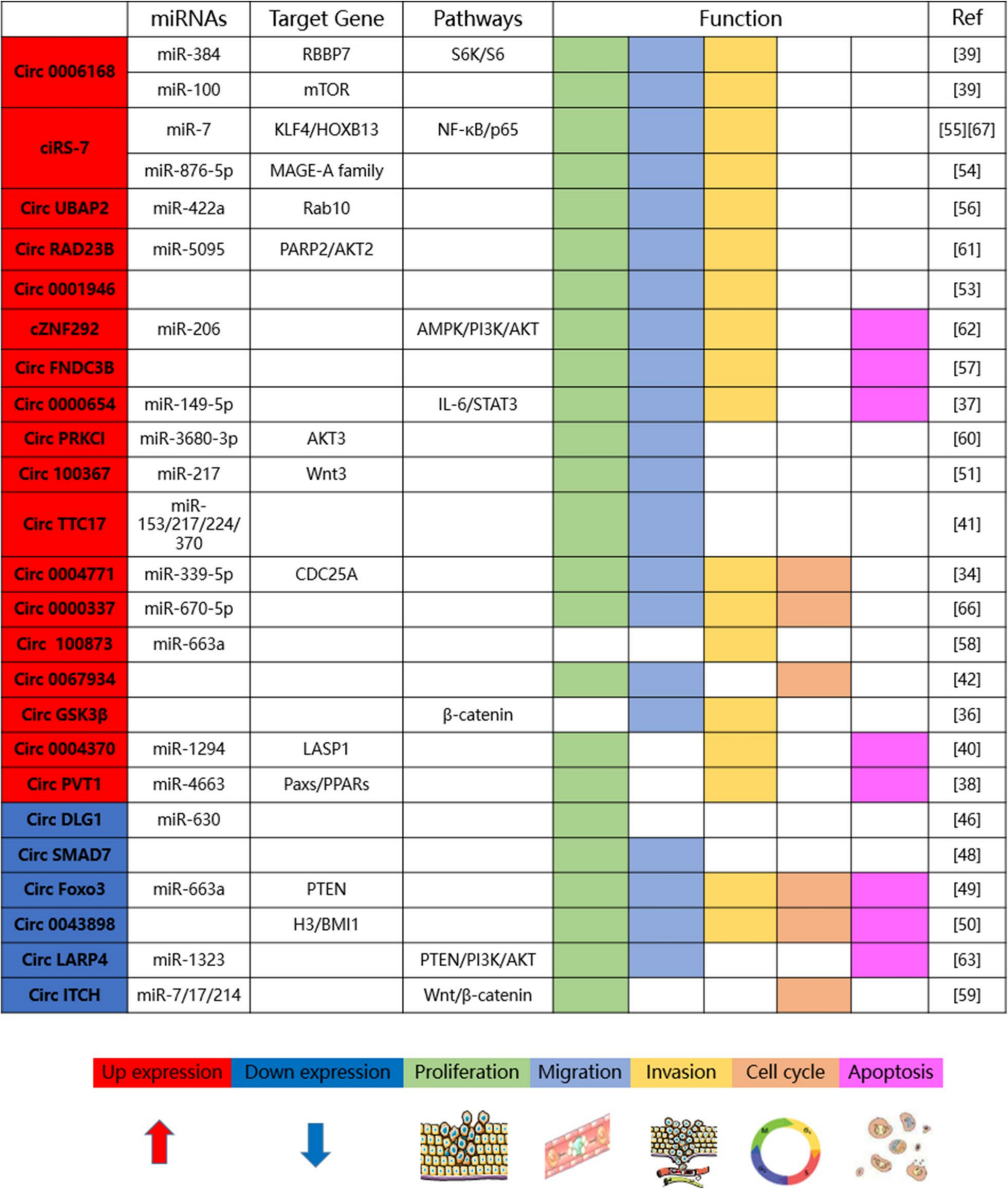
Roles of circRNAs in esophageal cancer. Red rectangles indicate up expression in cancer cells. Blue rectangles represent down expression. CircRNAs are involved in esophageal cancer cell proliferation, migration and invasion and are also associated with cell cycle and apoptosis through different mechanisms

As a new category of ncRNAs, the research on the role of circRNAs in the development of EC is still in its infancy. However, more and more evidences show that circRNAs plays an important role in the biological development of EC, such as regulating cell proliferation and apoptosis. For example, studies have found that the expression of ciRS-7 is up-regulated in ESCC, and ciRS-7 contains 19 miR-876-5p binding sites. Through the action of miRNA sponge, the tumor inhibitory activity of miR-876-5p is weakened and the expression of MAGE-A family of downstream targeted tumor antigens of miR-876-5p is enhanced. It is proved that the overexpression of ciRS-7 promotes the proliferation, migration and invasion of ESCC cells [54]. The expression of hsa_circ_0004370 was up-regulated in EC tissues and cell lines. Lost the function of hsa_circ_0004370 by siRNA significantly inhibited the proliferation and invasion of EC cells, and promoted cell apoptosis. Using bioinformatics methods, hsa_circ_0004370 can sponge miR-1294 and indirectly up-regulate the levels of LIM and SH3 domain protein 1 (LASP1), suggesting that hsa_circ_0004370 may be an oncogene affecting proliferation, apoptosis and invasion through the miR-1294/LASP1 axis [41]. Another study found that hsa_circ_0000654 was significantly up-regulated in ESCC tissues and cell lines, and its high expression was significantly related to increased T stage and local lymph node metastasis in patients with ESCC. Circ_0000654 is the sponge of miR-149-5p, which promotes the proliferation, migration, invasion and apoptosis of EC cells by indirectly activating IL-6/STAT3 signal pathway [38]. Recent studies have found that the expression of Circ UBAP2 was up-regulated, which promoted the proliferation, migration and invasion of ESCC. Further studies on the mechanism have proved that circ UBAP2 might play the role of oncogenes by regulating the miR-422a/Rab10 axis of ESCC [55]. Compared with paracancerous tissues, the expressions of hsa_circ_0067934, circFNDC3B, circ-TTC17, hsa_circ_0006168, circPVT1 and hsa_circ_100873 were significantly up-regulated in EC tissues [39, 40, 42, 43, 56, 57]. So far, cir-ITCH, circ-PRKCI, circRAD23B, cZNF292, circ-Foxo3, circGSK3 β, circLARP4 and hsa_circ_0004771 have been shown to regulate the proliferation and apoptosis of EC cells through different signal pathways [35, 37, 50, 58–62]. In a word, different results suggest that circRNAs may promote or inhibit development of EC through different mechanisms (Fig. 2).

### CircRNAs regulate EMT and metastasis in EC

In the process of EMT, epithelial cells lose polarized tissue and gain the ability of migration and invasion [63]. The abnormal activation of EMT promotes the invasion and spread of tumor cells, which is a necessary cellular process of tumor metastasis. Therefore, understanding and targeting circRNA to inhibit EMT and tumor metastasis is a potential process of inhibiting the malignant progression of EC.

**Fig. 3:**
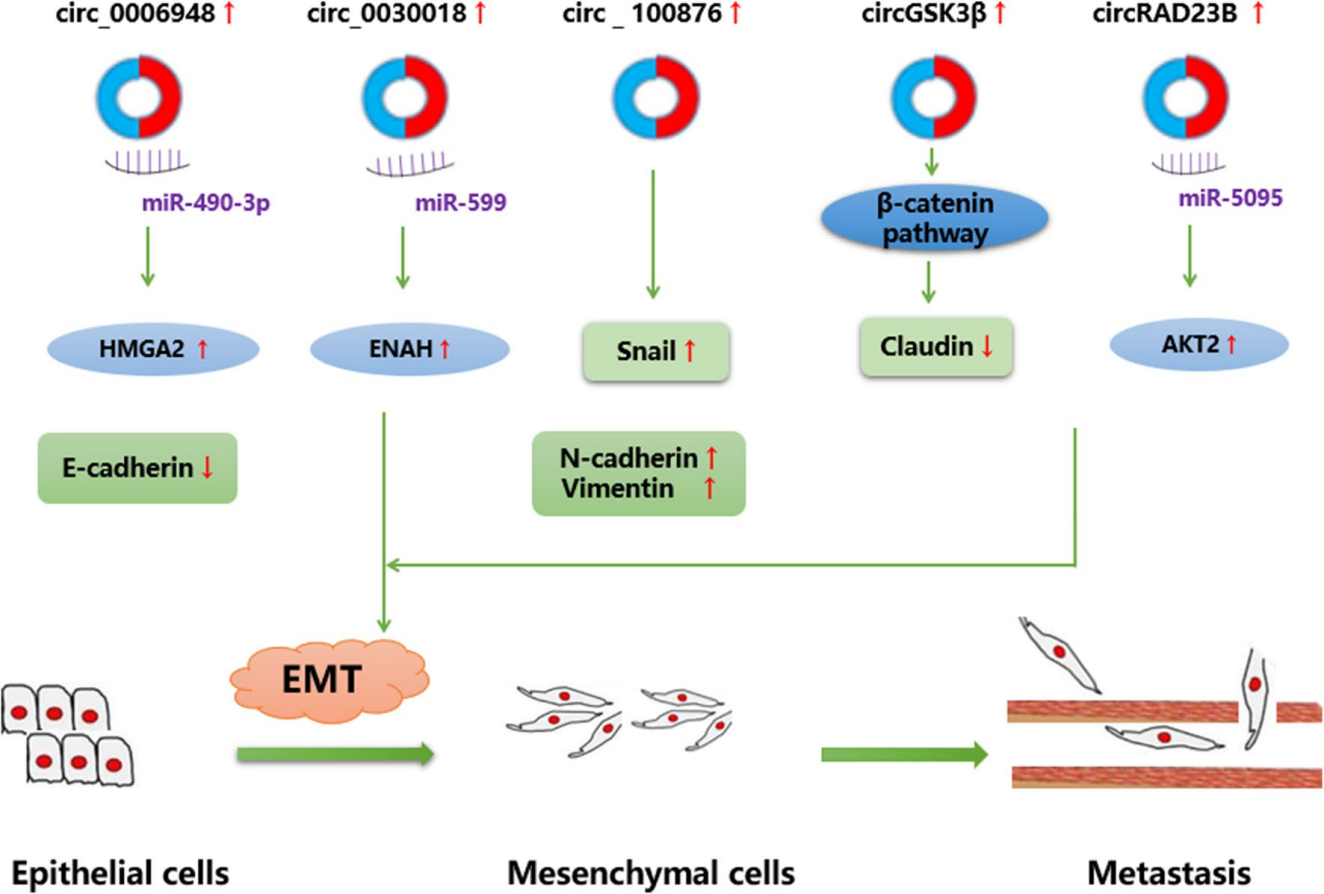
Interrelated regulatory of CircRNA and EMT. The biological role of circRNA in EMT and major interconnected regulatory networks in esophageal cancer. Several fundamental effectors including Vimentin, Cadherin, Snail and Claudin in this regulatory network. Some circRNAs via interacting with various target genes (including HMGA2, ENAH and AKT2) and β-catenin pathway promote EMT and initiate metastasis

It has been found that the up-regulated expression of hsa_circ_0006948 in ESCC is related to lymph node metastasis and poor prognosis. In addition, further studies have shown that hsa_circ_0006948 promoted the proliferation, migration and invasion of through sponging miR-490-3p, and induced the formation of EMT in EC cells [44]. Similarly, it is reported that the expression of circRAD23B [60], hsa_circ_0030018 [45] and circRNA_100876 [46] in EC tissues is up-regulated, which may promote the proliferation, migration and invasion of cancer cells by activating EMT in EC [45, 46, 60]. Therefore, it is necessary to seek tumor suppressor genes that inhibit EMT pathway and tumor metastasis, so as to delay the malignant progression of EC (Fig. 3).

### CircRNAs influence treatment resistance in EC

At present, in the treatment strategy of EC, chemotherapy, chemoradiotherapy and esophagectomy are the main treatment methods, but due to the emergence of drug resistance, some patients would have local recurrence and distant metastasis, leading low 5-year survival rate. Acquired radioresistance during radiotherapy is considered to be the most important cause of tumor local recurrence or treatment failure. Understanding the regulatory mechanism of circRNA involved in chemoradiotherapy resistance can identify new targets to optimize treatment.

The evaluation of circRNAs found that among the 3752 candidate circRNA genes detected, 57 circRNAs were up-regulated and 17 circRNAs were down-regulated in the radioresistance EC cell line (KYSE-150R) compared with the radiation-sensitive ESCC cell line (KYSE-150). Studies have shown that radioresistance EC cells are related to the abnormal regulation of circRNA. Most of their target genes are enriched in the Wnt signal pathway, indicating that these circRNAs play a regulatory role in EC resistance through the Wnt signal pathway. The differential expression of circRNA in EC, especially circRNA_001059 and circRNA_000167, may be related to the radioresistance of EC cells and affects the treatment and prognosis of EC [53]. The latest study found that the high expression of circRNA_100367 was related to the radiosensitivity of ESCC. Higher expression and potency of EMT was found in KYSE-150R than in KYSE-150. Silencing circRNA_100367 inhibited the proliferation and migration of KYSE-150R cells and reduced the expression of β-catenin, an important molecule of Wnt pathway, in KYSE-150R cells. In addition, circRNA_100367 bound to miR-217, and miR-217 targeted Wnt3 to enhance the radioresistance of KYSE150R cells through miR217/Wnt3 pathway. In vivo, circRNA_100367 silently suppressed the growth of KYSE150R cells under radiation. Therefore, circRNA_100367 reduced the radioresistance of EC cells through the miR-217/Wnt3 pathway, which would provide a potential target for reducing radiotherapy failure in patients with ESCC [52]. With the further study of drug resistance of tumor cells to chemoradiotherapy, circRNA, as a new biomarker, has great potential in predicting the efficacy and prognosis of chemoradiotherapy or interfering with clinical tumor therapeutic targets such as chemoradiotherapy.

## Conclusion

With the continuous progress in the field of RNA, circRNAs has become a new research hotspot. In recent years, our research has deepened our understanding of circRNA, from “transcriptional noise” to functional regulatory molecules that mediate different physiological and pathological processes. Its interaction with tumor has gradually attracted people’s attention. In this review, we mainly introduce the function of circRNAs and its as clinical biomarkers for the diagnosis, treatment and prognosis of EC, and further explore the role and significance of circRNAs in EC. Other unknown circRNA functions and potential biomarkers need to be studied in the future. Although circRNAs has made some progress in other human tumors, the research on the regulation of EC by circRNAs is still in its infancy, and some specific mechanisms of circRNAs-mediated EC are still unclear. Further study on the detailed mechanism of circRNAs regulating EC may provide new insights into how circRNAs can enhance or inhibit the occurrence and development of tumor, increase the research on the pharmacodynamics and safety of circRNA in the treatment of EC, and realize the clinical application of circRNAs as soon as possible.

## Data Availability

The datasets used and/or analyzed during the current study are available from the corresponding author on reasonable request.

## Abbreviations

circRNA: Circular RNA
EMR: Endoscopic mucosal resection
miRNA: microRNA
EC: Esophageal cancer
ESCC: Esophageal squamous cell carcinoma
EAC: Esophageal adenocarcinoma
ncRNA: Non-coding RNA
lncRNA: Long-chain non-coding RNA
pre-Mrna: Precursor mRNA
ceRNA: Competitive endogenous RNA
RISC: RNA-induced silencing complex
SRY: Sex-determining region Y
IRES: Internal ribosome entry site element
CEA: Carcinoembryonic antigen
EMT: Epithelial–mesenchymal transition
